# Growth factor release by vesicular phospholipid gels: in-vitro results and application for rotator cuff repair in a rat model

**DOI:** 10.1186/s12891-015-0542-1

**Published:** 2015-04-10

**Authors:** Stefan Buchmann, Gunther H Sandmann, Lars Walz, Thomas Reichel, Knut Beitzel, Gabriele Wexel, Weiwei Tian, Achim Battmann, Stephan Vogt, Gerhard Winter, Andreas B Imhoff

**Affiliations:** Department of Orthopaedic Sports Medicine, Klinikum rechts der Isar, Technical University of Munich, Ismaningerstr., 81675 Munich, Germany; Department of Traumatology, Klinikum rechts der Isar, Technical University of Munich, Ismaningerstr. 22, 81675 Munich, Germany; Clinical Trial Unit, University Hospital Basel, Schanzenstr. 55, Basel, Switzerland; Department of Experimental Oncology, Klinikum rechts der Isar, Technical University of Munich, Ismaningerstr. 22, 81675 Munich, Germany; Department of Pharmacy, Pharmaceutical Technology and Biopharmaceutics, Ludwig Maximilians University, Butenandstr. 5-13, 81377 Munich, Germany; Institute for Pathology and Cytodiagnostics, Urselerstr. 33, 61348 Bad Homburg, v.d.H Germany; Clinic for Orthopaedic Sports Medicine and arthroscopic Surgery, Orthopaedic Hospital Hessing Stiftung, Hessingstraße 17, 86199 Augsburg, Germany

**Keywords:** Growth factors, Controlled drug release, Rat model, Rotator cuff, Biomechanics

## Abstract

**Background:**

Biological augmentation of rotator cuff repair is of growing interest to improve biomechanical properties and prevent re-tearing. But intraoperative single shot growth factor application appears not sufficient to provide healing support in the physiologic growth factor expression peaks. The purpose of this study was to establish a sustained release of granulocyte-colony stimulating factor (G-CSF) from injectable vesicular phospholipid gels (VPGs) in vitro and to examine biocompatibility and influence on histology and biomechanical behavior of G-CSF loaded VPGs in a chronic supraspinatus tear rat model.

**Methods:**

G-CSF loaded VPGs were produced by dual asymmetric centrifugation. In vitro the integrity, stability and release rate were analyzed. In vivo supraspinatus tendons of 60 rats were detached and after 3 weeks a transosseous refixation with G-CSF loaded VPGs augmentation (n = 15; control, placebo, 1 and 10 μg G-CSF/d) was performed. 6 weeks postoperatively the healing site was analyzed histologically (n = 9; H&E by modified MOVIN score/Collagen I/III) and biomechanically (n = 6).

**Results:**

In vitro testing revealed stable proteins after centrifugation and a continuous G-CSF release of up to 4 weeks. Placebo VPGs showed histologically no negative side effects on the healing process. Histologically in vivo testing demonstrated significant advantages for G-CSF 1 μg/d but not for G-CSF 10 μg/d in Collagen III content (p = 0.035) and a higher Collagen I/III ratio compared to the other groups. Biomechanically G-CSF 1 μg/d revealed a significant higher load to failure ratio (p = 0.020) compared to control but no significant differences in stiffness.

**Conclusions:**

By use of VPGs a continuous growth factor release could be obtained in vitro. The in vivo results demonstrate an improvement of immunohistology and biomechanical properties with a low dose G-CSF application via VPG. The VPG itself was well tolerated and had no negative influence on the healing behavior. Due to the favorable properties (highly adhesive, injectable, biocompatible) VPGs are a very interesting option for biologic augmentation. The study may serve as basis for further research in growth factor application models.

## Background

Rotator cuff tears are a common cause of shoulder pain, reduced function and weakness of the arm in the elderly patient [1]. The retear rate still ranges between 25% and 94%, despite biomechanically proven stability of the primary tendon reinsertion [2-5]. Besides individual patient factors like age, tear size and tendon-/muscle quality the biological healing response is identified as essential factor to improve tendon healing [6]. Therefore current research is focusing on the use of growth factors (GF) for biological augmentation [7]. Although a number of studies using growth factors have been published they mostly used a single injection or a single intraoperative application at time zero. The reported limited success might be due to their very short in vivo half-life time and the previously described GF peak expression in tendon healing which is GF dependent between day 7 and 14 after injury [8,9]. To gain this biological activity at their physiological peak expression multiple GF injections or sustained GF release from a carrier is required. Multiple injections are invasive and include the risk of variation of the application site so that the sustained release remains a valuable option.

A previous study has shown that continuous release of G-CSF via an osmotic pump positively affected the supraspinatus tendon (SSP) remodeling in a chronic SSP tear rat model [10]. Nevertheless, the rate of pump dislocations (30%) and the invasiveness of the surgical procedure (subcutaneous pump implantation as foreign body and additional subacromial tube) were seen as significant disadvantages compromising the results obtained and can only be considered as a preclinical tool to establish the proof of principle. This led to the idea of an injectable vesicular phospholipid gel (VPGs) as a carrier for GF delivery. These gels are composed of nontoxic excipients, namely lecithin and aqueous buffer solution and show an excellent biocompatibility. The formulations are very robust and easy to produce, but allow on the other hand a burst free, continuous release of GFs over days and weeks, depending on their specific composition and the nature of the incorporated drug [11,12]. The easy application of such semi-solid, ointment like gels in open and also arthroscopic surgery, as well as the adaptation to different surface modalities due to the tunable mechanical properties of the gel formulation can be seen as further advantages. In vitro studies revealed the feasibility of continuous erythropoietin (EPO) release via such vesicular phospholipid gels over more than 400 h [13].

The purpose of the present study was to evaluate the in vitro release kinematics of G-CSF by phospholipid gels and in the following to examine the effect of continuous G-CSF release via phospholipid gels on tendon remodeling in a chronic supraspinatus tear rat model.

According to the purpose of the study we evaluated the hypothesis that a continuous G-CSF release by phospholipid gels could be achieved and that the application of G-CSF loaded VPGs as biological augmentation in a chronic supraspinatus rat model results in a significant improvement of histological and biomechanical healing properties.

## Methods

**Figure 1:**
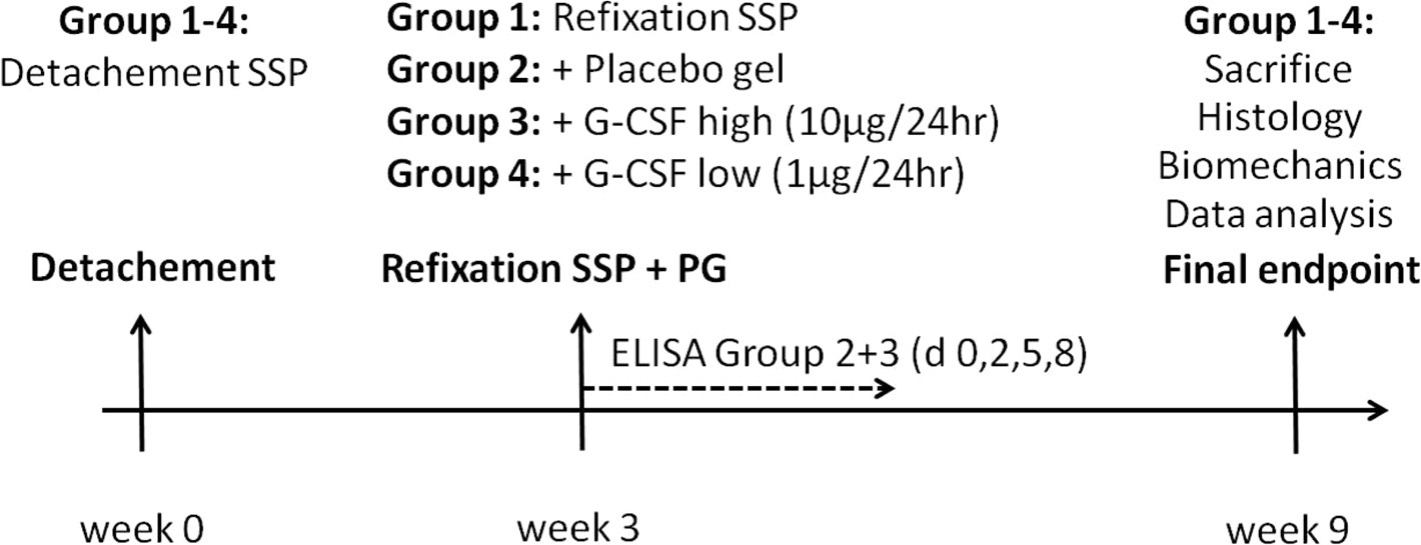
**Overview of the experimental set-up.**

In a first step, the aim was to evaluate in vitro the best combination of the gel’s release properties and viscosity for optimized intraoperative handling, therefore different variation of pH, G-CSF and lipid content were analyzed. Second the in-vivo effects of this developed gel were evaluated in a chronic tendon rat model. The systemic effects were analyzed direct postoperatively by G-CSF ELISA and 6 weeks postoperatively by histological and biomechanical testing (Figure 1).

### Vesicular phospholipid gel composition and in vitro release

The preparation of VPGs by dual asymmetric centrifugation (DAC) was based on the method described by Massing et al. [14]. In brief, the protein solution consisting of G-CSF (Neupogen, Amgen, Thousand Oaks, CA, USA), in 10 mM acetate buffer (pH 3.9 containing 0.004% Tween and 5% sorbitol) was added to the lipids (Egg phosphatidylcholine E80, Lipoid GmbH, Ludwigshafen, Germany). The mixture was homogenized in DAC by multiple runs of 1.5 minutes (to a total mixing time of 45 minutes) at a process speed of 3500 rpm. The samples were cooled at 2-8°C at interrupts (after 6–10 runs) to control the temperature of the formulation below 40°C.

The integrity and stability of G-CSF was analyzed by non-reducing SDS-PAGE (sodium dodecyl sulfate polyacrylamide gel electrophoresis) with subsequent silver staining. In vitro release tests were conducted within flow through cells based on the design described by Tardi et al. [15] at 37°C. PBS (phosphate buffered saline) buffer pH 7.4 (20 mM) was used as acceptor medium. The released G-CSF from VPGs was extracted from the release buffer by applying DMF (N, N-Dimethylformamide) and quantified by RP-HPLC (reversed phase high-performance liquid chromatography).

The influence of the lipid content on the specific release behavior of G-CSF VPGs was first investigated. VPGs were prepared with various lipid (egg PC, LIPOID E80) contents ranging from 400 mg/g to 490 mg/g. The concentration of G-CSF was 4 mg/g in all these formulations. In a second step, the influence of the G-CSF content on the release behavior of the system was investigated. VPGs were prepared with various concentrations of G-CSF (4 mg/ml and 8.4 mg/ml). The lipid content in these VPG-formulations was 450 mg/g (egg PC, LIPOID E80) (Table 1).

**Table 1:** **In-vitro G-CSF Release**

**Formulation (1 mg)**	**G-CSF VPGs_1**	**G-CSF VPGs_2**	**G-CSF VPGs_3**	**G-CSF VPGs_4**
Lipid content	400 mg/g	450 mg/g	490 mg/g	450 mg/g
G-CSF content	4.0 mg/g	4.0 mg/g	4.0 mg/g	8.4 mg/g
Initial release (Day 1)	64 μg	32 μg	43 μg	49 μg
Daily release amount range	56 μg-388 μg	32 μg-204 μg	32 μg-150 μg	25 μg-76 μg
Average release amount per day	149 μg	88 μg	89 μg	43 μg
Daily release percentage of the incorporated protein/%	3.7%	2.2%	2.2%	0.5%

Daily release of G-CSF from 1 g VPGs based on various compositions.

For the sterile production of the VPGs the protein solutions were sterile filtered and added to the previously sterilized lipid (gamma radiated) in the sterile sample containers (25 ml, PP) under aseptic conditions. From that point on, aseptic production was achieved using the hermetically sealed sample containers for the asymmetric centrifugation processes. The prepared gels were then transferred into 1 ml syringes (B. Braun Melsungen AG, Germany) under aseptic conditions and were implanted within 3 days after production.

### Application of growth factor loaded gels in a chronic supraspinatus tendon model

Sixty healthy (14 weeks old; 400 g) Sprague–Dawley rats were obtained by Charles Rivers (Sulzfeld, Germany) and included into the study. All animals received care in compliance with the guidelines of the local animal care committee following National of Institute of Health guidelines. The supraspinatus tendon (SSP) was completely detached and a transosseous refixation with additional VPG augmentation was performed after 3 weeks as described before [16] (Figure 1).

In short at primary surgery the supraspinatus tendon of the right shoulder was completely detached from its insertion site [17]. The rats were allowed unrestricted cage activity. For three days postoperatively the rats received weight adapted pain medication (metamizole oral and buprenorphine subcutaneous) every 12 hours. For repair of the chronic degenerated tendon at 3 weeks the previous scar was re-incised and a t-shaped delta incision was performed. Applying humero-acromial extension the marking suture was retrieved and the tendon was mobilized. All adhesions were released and the SSP tendon was refixed transosseously according to the technique described by Thomopoulos et al. [16]. In all cases, extensive subacromial mobilization allowed a repair without undue tension. Footprint and tendon surface were covered with in total 0.2 ml VPG. The 4 groups (each n = 15) consisted of the Control (no VPG), the Placebo (VPG without G-CSF), G-CSF high (10 μg/24 hr) and G-CSF low (1 μg/24 hr) (Figure 1). Finally the flaps of the T-incision were refixed transosseously to the acromion. The following wound closure and postoperative treatment was performed accordingly to the first operation. All animals recovered well from both surgeries. They were housed in cages with 4 to 6 rats and they were followed for 6 weeks after the second surgery. Food and water were supplied ad libitum.

### Systemic concentration of G-CSF

In order to assess the protein concentration in the serum/plasma samples, G-CSF was assayed by the enzyme-linked immunosorbent assay (ELISA Quantikine DCS50, R&D Systems, Minneapolis, MN, USA), designed to measure G-CSF in cell culture supernates, serum, and plasma. It contains E. coli-expressed recombinant human G-CSF and antibodies raised against the protein. It has been shown to accurately quantitate recombinant human G-CSF without obvious cross reactivity with rabbit serums [18].

Blood samples (2 μl) of the G-CSF high group (Release rate: 10 μg/24 hr) and placebo group were taken from the contralateral subclavian vein following short anesthesia with Isoflurane on day 0, 2, 5 and 8. The detailed steps of the assay followed the manual by the supplier.

### Histology and immunohistochemistry

All rats were euthanized 6 weeks after the SSP reconstruction. For n = 9 in each group the musculotendinous unit of the supraspinatus was exposed and removed proximally from the supraspinatus fossa and distally from the bony insertion of the humeral head for histological analysis and stored in 4% buffered formalin solution [17]. For analysis 5 μm sections from each group and both shoulders were stained with hematoxylin and eosin (H&E). The sections were cut parallel to the tendon fibers. A specialized pathologist then evaluated the sections were then qualitatively in a blinded fashion. Microscopy was performed on a digital microscope (Leica Microsystems, Jena, Germany) and image acquisition and analysis using a digital camera system (Nikon Inc., Duesseldorf, Germany). Six high magnified fields were analyzed per tendon cross-section to determine the proportion of degeneration, cell number and chronic or florid inflammation within the probes. For evaluation of tendon degeneration a modified MOVIN Score was used [19]. The subcategories (fiber structure, fiber arrangement, rounding of the nuclei, regional variations in cellularity, increased vascularity and hyalinization) were scored between 0 and 3, with 0 being normal, 1 slightly abnormal, 2 abnormal, and 3 markedly abnormal. The results of the subcategories and the score sum were evaluated. For immune histological assessment six high magnified fields were analyzed per tendon cross-section to determine the proportion of staining. A semi quantitative score ranging from 0 (no staining) to 3 (intense staining) was used for evaluation of treated and control shoulders. The results of each group and inter individual differences between treated and control shoulders were evaluated. The contribution of collagen was described with a collagen I/III quotient of the semiquantitative staining score and compared in a sole descriptive manner.

### Biomechanical testing

**Figure 2:**
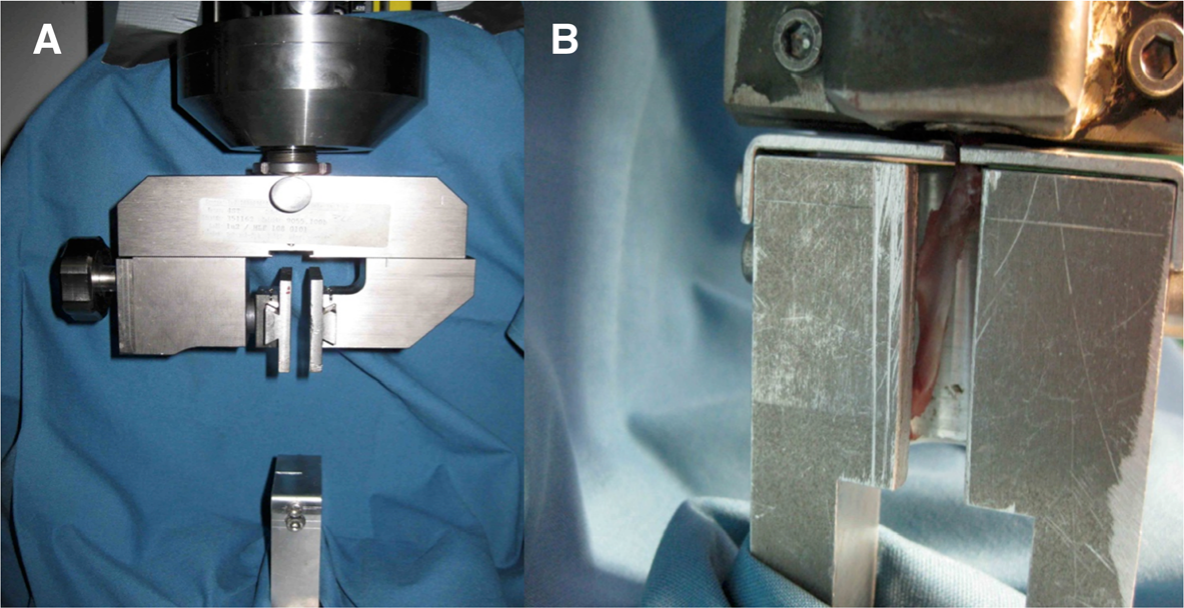
**Biomechanical testing setup. A)** Ball-beared mounting clamp for fixation of the cryoclamp in a mechanical testing machine (Zwicki 1120, Fa. Zwick) **B)** Rat supraspinatus tendon press-fixed in a cryoclamp and humerus placed in a mounting grid with bony humeral fixation for load to failure testing.

After scarification, the complete supraspinatus muscle was resected from the scapula in toto in 6 rats of each group and the humero-ulnary joint was disarticulated. Each specimen was frozen at −18°C separately and slowly thawed under room temperature for testing. During testing the thawed supraspinatus tendon was constantly moistened with sprayed isotone solution of sodium chlorid to anticipate drying out. Subsequently the proximal end (musculotendinous junction) was press-fixed in a cryoclamp while the humerus was placed in a mounting grid with bony humeral fixation (Figure 2A). Testing was performed with the shoulder at 90° of abduction. Cooling down the cryoclamp with 5 ml of liquid nitrogen (same amount on both sides of clamp) caused freezing of the clamp and the musculotendinous junction for rigid fixation, without affecting the free tendon (room temperature). The tendons were then mounted onto a mechanical testing machine (Zwicki 1120, Fa. Zwick) (Figure 2B). The construct was initially set to a pre-load of 0.1 N straightening and adjustment of each tendon. A dynamic preconditioning in 10 cycles with a speed of 0.2 mm/min between 0.1 and 0.5 N was performed. Five seconds after preconditioning the specimen was axially pulled at a constant speed of 10 mm/min until maximum load to failure [20]. Simultaneously contralateral tendons were tested in the same way for further investigation and percentage statistical analysis. Stiffness [N/mm] and ultimate failure load [N] were calculated with SPSS software (SPSS v12.0; SPSS, Chicago, Illinois).

### Animal ethics statement

The study-protocol was approved by the local government (Regierung von Oberbayern, Munich, Germany, N° 55.2-1-54-2531-138-07, 09.12.2009).

### Statistical analysis

Between group differences (MOVIN) were compared using Wilcoxon Test (Software R, Version 2.10.0). Statistical significance was set at p ≤ 0.05. A pretesting power-analysis (power > 0.80) was performed for the sum score (MOVIN) with a predicted score difference of 3 points and a SD of 2.25 points. The biomechanical properties of the tendons were analyzed across treatments with use of analysis of variance with the level of alpha set at 0.05. Pairwise comparisons were performed with use of two-tailed paired t tests. Statistical analysis was performed with use of SPSS software (SPSS v12.0; Chicago, Illinois, USA).

## Results

### Vesicular phospholipid gel composition and in vitro release

**Figure 3:**
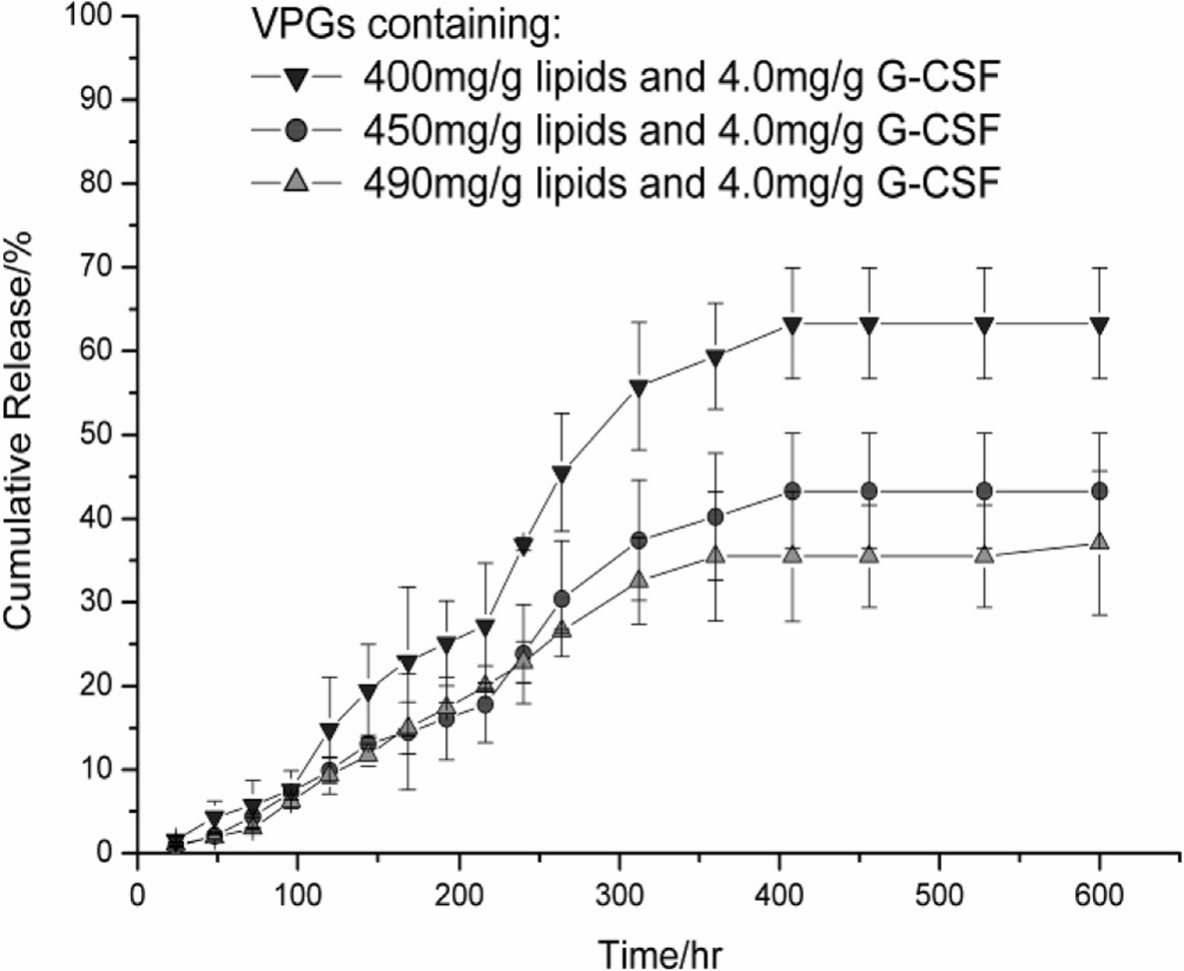
**Cumulative release of G-CSF from VPGs based on various concentrations of lipids (egg PC, LIPOID E80) (Mean ± standard deviation, n = 3 each group).**

No detrimental effect of the preparation process in a dual asymmetric centrifuge on the quality of G-CSF was found when analyzed by SDS-PAGE. The in-vitro release studies clearly demonstrated the potential of VPGs for sustained delivery of G-CSF. It was observed that G-CSF was released from all formulations in a linear manner following zero-order kinetics. Increasing the lipid content from 400 mg/g to 450 mg/g resulted in a slower release rate. However, the VPG-system appeared rather robust when the lipid content was changed from 450 mg/g to 490 mg/g, i.e. the release rate was not much affected. For VPGs based on 400 mg/g lipids and 4.0 mg/g G-CSF 63% of the total entrapped protein was delivered over 408 hrs whereas VPGs based on 450 mg/g lipids delivered 43% and VPGs with 490 mg/g lipids delivered 35% within the same period (Figure 3).

The release rate for the in vivo animals study had to be adjusted. We assumed a daily release rate of a total of 1 μg/24 hours and 10 μg/24 hours per animal, respectively, to be adequate for the desired biological effect. A total volume of 200 μl of gel should be applied. With that specification in mind, formulations with 0.2 mg/g and 2.0 mg/g G-CSF were prepared. The average release rates for the previously tested gels and the calculated, expected release rates (over about 400 hours) for the preclinical batches are provided in Tables 1 and 2.

**Table 2:** **VPG-Formulations**

	**G-CSF**	
Buffer in protein solutions	10 mM acetate buffer pH 3.9	
	0.004% Tween	
	5% sorbitol	
Protein concentration per ml VPGs	200 μg	2 mg
Lipid content per ml VPGs	450 mg/g	
Expected release amount pro 24 h	1 μg	10 μg

Formulations of VPGs, loaded with various contents of growth factors.

The final product combined the required properties of the daily release rate (1 μg/24 hours respectively 10 μg/24) and the viscosity to allow to inject the gel or also to use it arthroscopically.

### Systemic concentration of G-CSF

**Figure 4:**
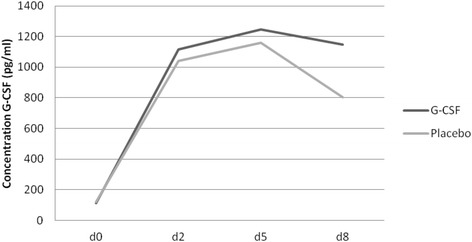
**Concentration of G-CSF (pg/ml) in the rat serum (d = days postop.): Comparison of G-CSF high (10 μg/24 h) and Placebo VPGs (p > .05 for all time points).**

The Quantikine G-CSF Immunoassay was validated for the quantification of G-CSF in the serum samples. No cross-reactivity of the assay was observed with the rat serum. The results revealed no significant differences within the first 8 postoperative days (d0 p = .444;d2 p = .440;d5 p = .307;d8 p = .219) between Placebo VPGs and G-CSF high (10 μg/24 h) (Figure 4).

### Macroscopic assessment

At time of sacrifice all tendons were intact including the transosseous suture. Between placebo and intervention groups no macroscopic difference could be found concerning hypertrophy of the tendon.

### Histology and immunohistochemistry

**Figure 5:**
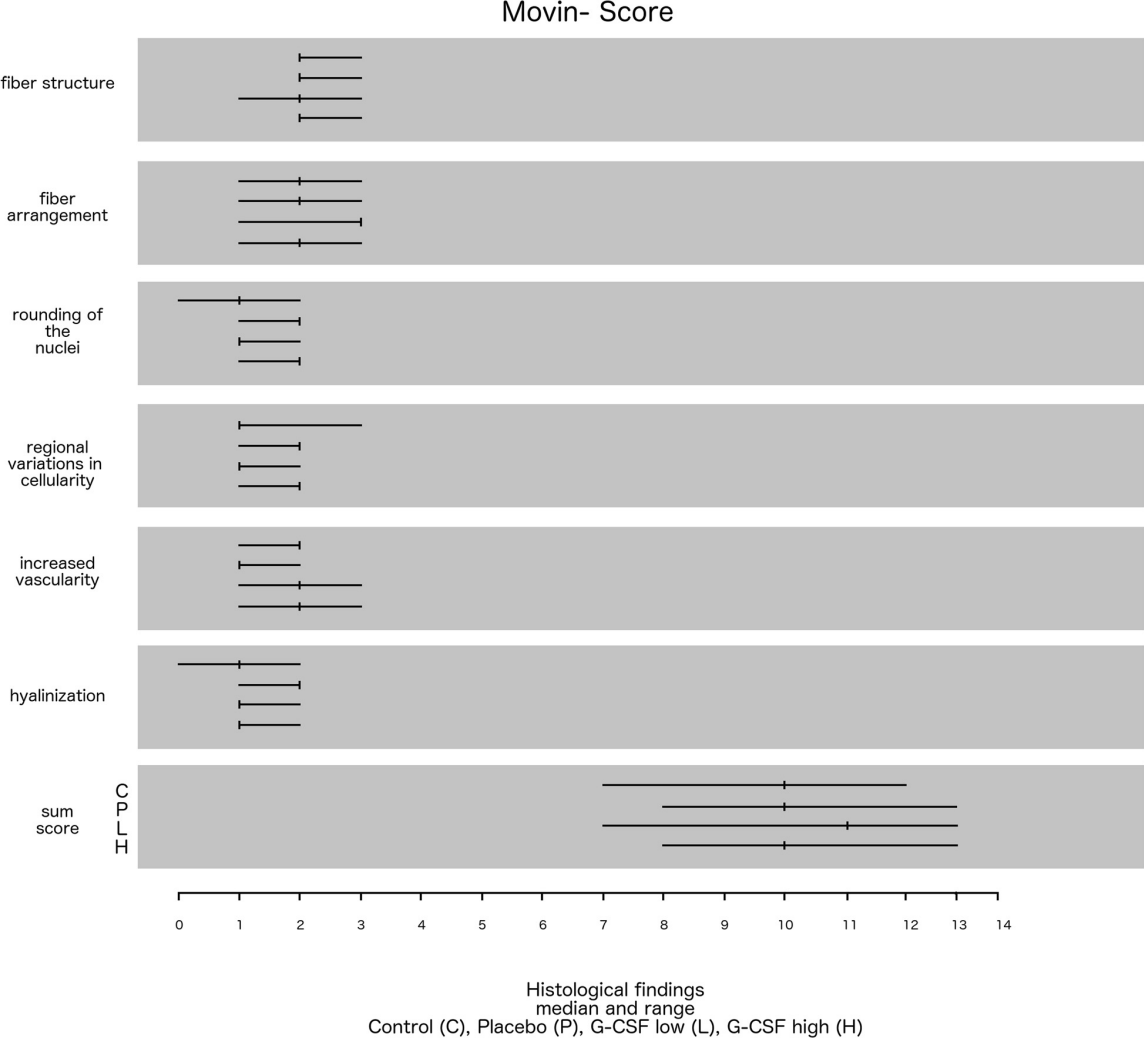
**MOVIN-Score: Median and range of subcategories and sum score (C = control; P = Placebo; L = G-CSF low; H = G-CSF high).**

The MOVIN sum scores showed no significant differences between all groups. Subcategory analysis revealed significant reduction of cellularity variation for G-CSF low and Control compared to Placebo (p = .009/p = .041). The Placebo group also showed a significant higher grade of hyalinization compared to control (p = .021). Detailed data is shown in Figure 5.

**Figure 6:**
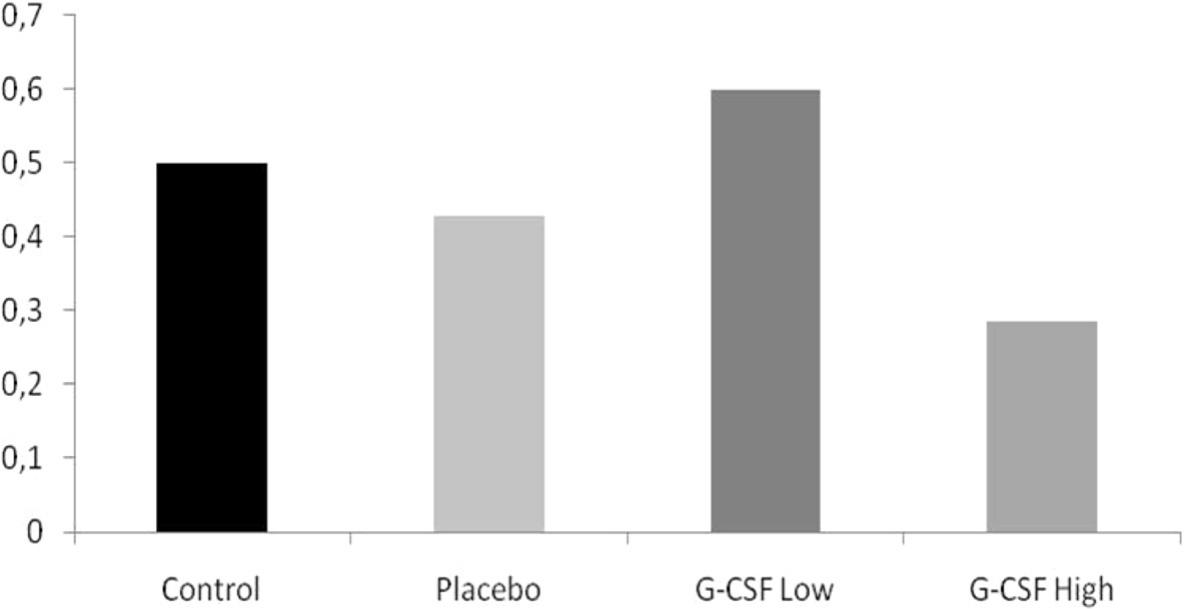
**Immunohistochemistry – Ratio Collagen I/Collagen III.**

For collagen I there was no significant intergroup difference. Collagen III staining revealed a significant lower level for Placebo (p = .003) and G-CSF low (p = .035) compared to the control group. G-CSF high showed a significant increased Collagen III content compared to Placebo (p = .004) and a tendency of a higher content compared to G-CSF low (p = .053). The Collagen I/III ratio was increased for G-CSF low (0.6) compared to Control (0.5), Placebo (0.42) and G-CSF High (0.29) (Figure 6).

### Biomechanical testing

Mean load to failure showed no significant differences between treatment groups. Compared to the healthy contralateral side all groups had a significant lower load to failure (Placebo p = .014; G-CSF low p = .042; G-CSF high p = .03). The load to failure ratio (treated/contralateral) revealed a significant better restoration of contralateral values for G-CSF low compared to Placebo (p = .020). For G-CSF high the difference was not significant (p = .062) (Table 3).

**Table 3:** **Biomechanical testing**

**Group**	**Mean load to failure [N]**	**Deficit to mean contralateral side [N]**	**Reduced load to failure to contralateral side = ratio [%]**	**p (compared to ratio Placebo)**
**Contralateral**	34.15 ± 9.1*	-	-	-
**Placebo**	21.40 ± 10.3	−16.78	62.71	-
**G-CSF Low**	26.19 ± 7.8	−5.68	81.62	0.020*
**G-CSF High**	24.78 ± 6.5	−7.63	80.39	0.062
**Group**	**Mean stiffness [N/mm]**	**Deficit to mean contralateral side [N]**	**Reduced stiffness to contralateral side = ratio [%]**	**p (compared to ratio Placebo)**
**Contralateral**	34.08 * ± 5.5	-	-	-
**Placebo**	18.68 ± 10.4	−20.78	47.34	-
**G-CSF Low**	16.57 ± 6.1	−17.76	48.27	0.242
**G-CSF High**	22.04 ± 7.0	−6.43	77.41	0.078

Load to failure (N) and Stiffness (N/mm) – mean and standard deviation (±) of absolute values and ratio to healthy contralateral side (%).

*Intergroup comparison showed significant differences only in comparison to the healthy contralateral side in load to failure (Placebo .007; G-CSF low .021;G-CSF high .015) and stiffness (p < .01 for all groups). Treatment group comparisons showed non significant differences (p > .05).

Measurement of stiffness revealed no significant differences between treatment groups. Compared to the healthy contralateral side all groups except G-CSF high had a significant lower stiffness (Placebo p = .033; G-CSF low p = .011; G-CSF high p = .102). The stiffness failure ratio (treated/contralateral) revealed a higher but not significant restoration of contralateral values for G-CSF high compared to Placebo (p = .078). For G-CSF low there was no difference (Table 3).

## Discussion

The hypothesis was proven partially as biomechanical testing of load to failure showed significant advantages for the G-CSF low but not for the G-CSF high group (1 μg/24 hr). Accordingly significant differences in immunohistology (absolute staining as well as the ratio of Collagen I/III staining) as a marker of tendon organization and maturation confirmed the positive effect of G-CSF low (1 μg/24 hr).

Additionally the previously described excellent biocompatibility of the VPGs was proven in the animal study as the placebo group showed no consistent significant difference in histology and biomechanics compared to the control group [11,12]. Also the macroscopic evaluation revealed no hypertrophic scar tissue or granuloma formation so that a usage for local applications of drug delivery in vivo can be envisaged without risk of negative side effects from the gel itself.

The in-vitro release studies provided clear evidence, that VPGs are a feasible scaffold for sustained delivery of G-CSF. G-CSF can be encapsulated by dual asymmetric centrifugation in vesicular phospholipid matrices without inducing protein destabilization. Furthermore, the phospholipid gel (VPG) enables sustained G-CSF release. In a wide range of formulation variables the release occurs in a quasi-linear manner without burst effect which is of particular advantage for indications that require constant drug levels over prolonged time periods. The desired release kinetics can be achieved by adjusting the lipid composition or the G-CSF loading amount. Both parameters were shown to affect the matrix erosion which in turn is the dominant mechanism controlling protein release [13].

In current literature, a vast number of growth factors were used in animal research on tendon repair [13,15,21-23]. Positive effects on tendon healing were reported for various GFs, but there is no clear data for a single GF with outstanding superior characteristics. Accordingly the choice of the used growth factors was based on data for improvement of tendon healing in animal trials, availability, and clinical experience [10,18,19,24-27]. One commercially available growth factor is G-CSF, which has been shown to affect the inflammatory response by direct activation of neutrophile granuclocytes [7,21]. In addition it promotes chemotaxis of mesenchymal stem cells and granulocytes and is involved in microvessel enhancement [28,29]. In an in vitro study Marmotti et al. [24] accelerated the outgrowth of chondrocytes which might be useful for the reorganization of the enthesis. Previously significant improvements of histological scores in a chronic SSP rat model by application of G-CSF via an osmotic pump over 20 days have been shown [10].

The application method should have a significant effect on the results of studies for biological tendon augmentation, since recent data estimate the time of the peak for physiological GF expression between week 1 and 2 after SSP refixation in animal models (rat/rabbit) [8,9]. Respecting this knowledge a programmed release of GF in close accordance to their physiologic release should be aspired. Sasaki et al. [27] showed positive effects on tendon-bone integration in ACL reconstruction (dog model) by using a continuous delivery of GCSF via a gelatin gel.

In the previous study G-CSF was used in a dose of 5 μg/day [10]. To ensure a significant dose difference in this study 1 and 10 μg G-CSF/day were chosen. In current literature local application of a single dose of 5 μg G-CSF and subcutaneous injections of 0.3 to 60 μm/kg per day for systemic treatment are described [24-27]. To our knowledge no study has been published so far investigating dose related effects of G-CSF in treatment of musculoskeletal disorders. The differences between the two groups in this study (1 and 10 μg G-CSF/day) and to the previous study (5 μg/day) might be explained by a dose related effect with adverse effects in a local overdose scenario. For different growth factors e.g. b-FGF an adverse effect for higher doses with hypertrophic tendon healing and reduced mechanical properties is described by Fukui et al. [30].

The accuracy of the release rate transferred from in vitro to in vivo highly depends on the local erosion situation, in this case the subacromial space, which is unpredictable and hard to simulate in vitro [11]. A standardized flow-through release cell with a rather slow flow rate and erosion force was used as previously described [13,15]. Together with the systemic data from the postoperative G-CSF ELISA no conclusion can be drawn about the actual local release rate because the results allow different interpretations. On the one hand an isolated local effect may not affect the dosage of G-CSF in the central-venous blood, on the other hand a local release of only minimal amounts of G-CSF below detection limit cannot be excluded. However, a systemic influence on venous blood G-CSF levels can be excluded.

### Limitations

In the current study, we performed a continuous subacromial GF application, i.e. we achieved a high growth factor dose also during the expression peaks. The manufacturing process of these phospholipid gels includes as well the disadvantage that for each protein the optimal composition (protein and lipid content) has to be tested separately. But that also allows adaptable release rates for different implementations. Due to the technical limitations of practicable slow release gel formulations, a physiological growth factor release profile could not be simulated and we do not know how this influences the tendon healing. The experiments were performed in a small animal model, which has been used in previous studies and shown to create a chronic supraspinatus tear, which is comparable to the tissue found in chronic human tendon tears. According to current literature, the rat model has been found superior to other animal models for rotator cuff pathologies but still some restrictions remain (self-healing potential, fatty infiltration) [17,31,32]. Additionally the application of 0.1 ml VPG on the footprint and 0.1 ml VPG on the reconstructed tendon in a small animal model involves the risk of variations of the local applied VPG volume due to differences in anatomy (size) and intraoperative bleeding. Furthermore the in-vivo study includes only one time point of testing after the SSP reconstruction (6 weeks after surgery). So we have no information about the GF effect in the early SSP healing phase and if a potential acceleration or deceleration of the SSP early healing phase would have occurred.

## Conclusions

By use of VPGs a continuous growth factor release could be obtained in vitro. The in vivo results demonstrate an improvement of immunohistology and biomechanical properties with a low dose G-CSF application via VPG. The VPG itself was well tolerated and had no negative influence on the healing behavior.
